# The Uses of Hospitals

**Published:** 1893-06-10

**Authors:** Edward Berdoe


### EDITOR'S LETTER-BOX.

[Our correspondents are reminded that prolixity is a great bar to publication, and that brevity of style and conciseness of statement greatly facilitate early insertion.]

### THE USES OF HOSPITALS.

SIR,—In your article on the Gains to Medicine which are due to our hospitals, you proceed on the assumption that these institutions mainly exist for the advancement of physiology and medical science, and you argue that the efficiency of the hospitals must be maintained with ever increasing generosity if the world desires to be strong, healthy, and energetic. With all respect I venture to suggest that this is not quite the way in which to urge the claims of our hospitals. The public at large—whatever may be the idea of the medical profession—consider that hospitals exist chiefly as charitable institutions for the cure of sick folk, and generous persons are induced to subscribe to them mainly from a desire to help their fellow creatures when stricken down with disease or by accident. The idea of the advancement of medicine, still less of physiology, enters but little, I imagine, into the motives which induce the charitable to send their cheques to the hospitals, and I believe great injury is done to the funds of such institutions by the idea that their chief uses are to educate doctors. I am quite sure that you do not desire to convey any such sentiment by your remarks, yet it must be admitted they are open to this objection. The only way, in my humble opinion to bring up the income of the hospitals to the highest point necessary for their efficiency is to disabuse the minds of the subscribers of the idea that education stands first, and charity only second in the minds of their promoters and officers.—I am, sir, yours, &c., EDWARD BERDOE, M.R.C.S., &c.

London, June 3rd, 1893.

*** We entirely agree with Mr. Berdoe as to the incomparable value of hospitals to the sick poor. But, if in addition to such value they are found to be almost equally valuable as subserving the general health and well-being of the whole population, we cannot but think that that is an additional reason for their liberal support; and we think also the public generally agrees with us in this idea.—ED. *T. H.*

